# From Cluster to Claim: Calibrating Interpretation in Single‐Cell Transcriptomics

**DOI:** 10.1002/ggn2.70045

**Published:** 2026-08-27

**Authors:** Shutong Lin, Guangchuang Yu

**Affiliations:** ^1^ Department of Bioinformatics School of Basic Medical Sciences Southern Medical University Guangzhou China

**Keywords:** biological interpretation, claim calibration, evidence strength, large language models, mechanistic inference, single‐cell transcriptomics

## Abstract

The widespread adoption of single‐cell transcriptomics has expanded our ability to study cellular heterogeneity and molecular states. However, the high‐resolution view it provides can also make it easier for descriptive data patterns to be translated into biological claims that go beyond the underlying evidence. We advocate a more calibrated approach to interpretation in single‐cell transcriptomic studies. We focus on two common points of vulnerability: the direct equation of computational clusters with biological cell types and the treatment of pathway enrichment as sufficient evidence for mechanistic conclusions. These examples are offered as illustrations rather than as a systematic survey of the field. We therefore propose a three‐tier logical framework—Observation, Inference, and Claim—to clarify the boundaries between statistical results, biological interpretation, and mechanistic claims. Single‐cell transcriptomics is powerful for hypothesis generation, state discovery, and heterogeneity profiling, but strong mechanistic claims still require orthogonal validation. As large language models (LLMs) are increasingly used in cell‐type annotation and biological narrative generation, explicit calibration between evidence strength and claim strength becomes even more important.

## 1 Introduction

Single‐cell transcriptomics has rapidly expanded the frontiers of biomedical research, enabling investigators to characterize cellular heterogeneity and molecular states with unprecedented resolution [1]. What was once a specialized laboratory approach has become a widely used strategy for transcriptome‐level profiling across diverse biological contexts. Advances in high‐throughput single‐cell transcriptomic profiling, standardized workflows, and mature analysis pipelines have lowered technical barriers [2, 3, 4]. This technological expansion has been highly productive, but it also raises an interpretive challenge: descriptive data patterns can be translated into biological claims that exceed the strength of the underlying evidence.

Historically, single‐cell transcriptomic studies were limited by the difficulty of detecting molecular patterns at cellular resolution [5, 6]. Today, the challenge is different. Common analytical outputs—including clustering, differentially expressed genes (DEGs), pathway enrichment, and pseudotime trajectories—can readily support biologically plausible narratives, but plausibility is not the same as validation. Clustering is a statistical representation of the data and may reflect developmental lineages, cell‐cycle effects, stress responses, sampling composition, batch effects, or technical noise [3, 4, 7]. Consequently, cell annotation should be treated as an inference supported by markers, references, controls, and biological context, rather than as a direct observational fact [7, 8]. Similarly, differential expression and pathway enrichment indicate molecular associations or functional potential, but they are insufficient on their own to establish causal mechanisms [9]. In short, higher‐resolution data do not automatically justify stronger biological claims.

As single‐cell transcriptomic results increasingly shape downstream hypotheses about cell identity, regulatory programs, and disease mechanisms, it becomes important to distinguish what the data show, what they plausibly suggest, and what has been mechanistically validated [10, 11]. This distinction is especially relevant as LLMs are increasingly used to assist cell‐type annotation, gene‐set interpretation, and biological narrative generation [12, 13]. Recent work further suggests that LLM‐assisted writing can inflate the strength of scientific claims, underscoring the need to check whether mechanistic language is supported by the underlying evidence [14]. In this Comment, we propose a practical framework for calibrating interpretation rather than another technical workflow. Specifically, we present an Observation‐Inference‐Claim framework and an accompanying Evidence Ladder to clarify when single‐cell transcriptomic results support descriptive statements, when they support biological interpretations, and when they justify stronger mechanistic claims.

## 2 Two Common Jumps in Single‐Cell Transcriptomic Interpretation

When biological narratives are constructed from single‐cell transcriptomic data, interpretive overreach often arises not from overt computational errors, but from subtle shifts in the logical status of the results. Statistical outputs may be written as if they were biological facts, and plausible interpretations may be elevated into mechanistic claims. The following two examples illustrate how this problem enters manuscript interpretation. They are not intended as a systematic assessment of how often these problems occur across the literature.

### 2.1 From Statistical Clusters to Biological Cell Types

A common point of vulnerability in scRNA‐seq analysis is the direct equation of computational clusters with biological cell types. Clusters visualized in dimensionality‐reduced space, such as UMAP or t‐SNE, are algorithmic representations of transcriptomic similarity rather than direct observations of naturally bounded biological entities [7]. A cluster may reflect lineage‐specific transcriptional programs, but it may also be influenced by cell‐cycle variation, dissociation‐induced stress, metabolic state, sampling composition, batch effects, ambient RNA, or other technical factors [3, 15, 16].

Cell‐type annotation should therefore be treated as an inference supported by evidence, not as a raw observational fact. This does not mean that annotation is arbitrary or unreliable. Substantial methodological work has improved annotation through curated marker sets, reference atlases, automated classifiers, reference‐based mapping, and strategies for representing uncertainty [3, 4, 17]. Well‐calibrated annotation is strongest when multiple lines of evidence converge, including canonical markers, reference mapping, sample‐level controls, tissue context, and, where possible, orthogonal validation such as protein expression or spatial localization.

A useful contrast is reference‐guided annotation of peripheral blood mononuclear cells (PBMCs) using multimodal single‐cell references. In such settings, major immune cell populations can be assigned with higher confidence when transcriptomic markers are concordant with surface‐protein measurements, curated reference labels, and established immune‐cell biology. For example, multimodal PBMC reference maps have shown that combining RNA profiles with antibody‐derived protein measurements improves the resolution and reproducibility of immune cell annotation [18]. The concern, then, is not annotation itself, but overconfident annotation based on clustering or marker enrichment alone.

The risk becomes apparent when cluster labels are assigned without sufficient attention to controls and technical confounders. Studies of droplet‐based scRNA‐seq have shown that ambient RNA contamination can introduce transcripts from one population into another, while barcode swapping can misassign reads across libraries [15, 16]. In infection studies, for example, an apparent cluster enriched for viral transcripts would require careful evaluation against negative controls, ambient RNA correction, and sample provenance before being interpreted as a true infected cell state. If a similar signal appears in uninfected controls, the more cautious interpretation is technical contamination or a library‐level artifact rather than a bona fide biological population. In other words, an apparently coherent signal can still arise from technical carryover if controls and sample provenance are not examined carefully. Thus, marker enrichment within a cluster can support a candidate annotation, but it is not by itself definitive proof of biological identity.

### 2.2 From Molecular Profiles to Functional Mechanisms

A second common jump occurs when descriptive molecular profiles are converted into causal or mechanistic conclusions. Single‐cell transcriptomic data provide high‐resolution measurements of gene expression, but higher‐resolution data do not automatically establish causal relationships. Differential expression, pathway enrichment, inferred regulatory programs, and pseudotime trajectories are powerful tools for hypothesis generation, yet they remain primarily descriptive or inferential unless supported by additional validation.

Differential expression characterizes differences in transcript abundance under a given condition. However, the transition from mRNA abundance to protein activity, metabolic flux, and cellular behavior involves translational regulation, post‐translational modifications, spatial organization, and context‐dependent signaling [19]. Similarly, pathway enrichment indicates molecular association or functional potential, but it does not by itself demonstrate that a pathway drives or mediates a biological process. Strong mechanistic verbs such as “drives,” “mediates,” or “is required for” are better reserved for settings in which causal direction, necessity, or sufficiency has been tested through perturbation, time‐resolved experiments, lineage tracing, or orthogonal functional assays [9, 20, 21].

Pseudotime analysis illustrates this distinction. Trajectory methods can order static transcriptomic profiles along a computational continuum and generate useful hypotheses about state transitions [22, 23]. However, without lineage tracing, live‐cell imaging, time‐series sampling, or perturbation, a pseudotime path should not be treated as direct evidence of a biological sequence of events [21, 22, 23]. Likewise, computational inference of gene regulatory networks can prioritize candidate regulators and increase the plausibility of regulatory relationships, but it does not by itself confirm causal regulation [24]. In these cases, the calibrated conclusion is that the data suggest candidate mechanisms for further testing, not that they establish mechanism.

LLMs may add a further layer to this interpretive problem because they are increasingly used to assist cell‐type annotation and biological narrative generation [12, 13]. Their fluent outputs may make descriptive gene lists and enrichment results appear more mechanistically coherent than the underlying evidence warrants. Recent work further suggests that LLM‐generated scientific summaries can overgeneralize research conclusions, underscoring the need to check whether mechanistic language is supported by the available evidence [14]. For single‐cell transcriptomic studies, LLM‐assisted text should therefore be evaluated not only for factual accuracy, but also for whether the strength of its claims remains aligned with the underlying evidence.

## 3 Framework: Separating Observation, Inference, and Claim

**FIGURE 1:**
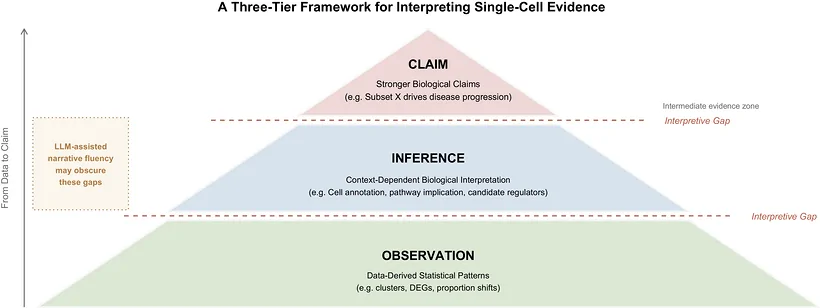
Observation‐Inference‐Claim framework for calibrating interpretation in single‐cell transcriptomic studies. The Observation layer (base) includes data‐derived statistical patterns, such as clusters, differentially expressed genes, enrichment scores, and pseudotime ordering. The Inference layer (middle) assigns provisional biological meaning to these patterns by integrating markers, reference datasets, tissue context, controls, and uncertainty assessment. The Claim layer (top) represents stronger biological conclusions, especially those involving causality, mechanism, or function. Dashed lines indicate interpretive checkpoints where additional evidence is needed before moving to a stronger level of conclusion. LLM‐assisted writing may obscure uncertainty around these checkpoints if fluent narrative generation is not accompanied by explicit evaluation of evidence strength.

One way to reduce interpretive overreach is to make the transition from data‐derived patterns to biological conclusions more explicit. We propose a three‐tier framework that separates Observation, Inference, and Claim (Figure 1). This framework is not meant to replace existing best‐practice guidance for single‐cell analysis. Instead, it addresses a narrower problem: how to keep conclusion strength in line with evidence strength when transcriptomic patterns are turned into biological claims.

### 3.1 Defining the Three‐Tier Hierarchy

The Observation layer refers to data‐derived statistical patterns generated by computational analysis. These include cluster structures in dimensionality‐reduced space, DEGs, enrichment scores, inferred trajectories, and other workflow outputs. Although such outputs are not independent of preprocessing choices, model assumptions, or parameter settings, they represent the least interpretive layer of the analysis [3, 7]. This layer primarily answers a simple question: what pattern is present in the data?

The Inference layer assigns provisional biological meaning to observed patterns by integrating computational results with prior biological knowledge, tissue context, reference datasets, appropriate controls, and where available, orthogonal evidence. For example, high expression of CD68 and ITGAM, together with other lineage markers, may support the annotation of a myeloid population based on established cell‐marker resources [25]. However, further describing the same population as “activated macrophages” requires additional contextual evidence, because macrophage activation states are heterogeneous and context‐dependent [26]. Inference should therefore be understood as a biologically plausible rather than a direct observation. The same transcriptomic pattern may support different interpretations across tissue niches, disease states, or experimental conditions [17].

The Claim layer represents a stronger step: it converts observations and inferences into broader biological conclusions. Claims often involve causality, mechanism, necessity, sufficiency, or functional consequence. Statements such as “a cell subset mediates immune evasion” or “a pathway drives disease progression” require evidence beyond transcriptomic association alone [9, 19]. They are strongest when supported by orthogonal validation, perturbation experiments, spatial or protein‐level confirmation, lineage tracing, or functional assays [18, 21, 27]. In this framework, the central question is not whether a claim is biologically plausible, but whether the available evidence is strong enough to support it.

### 3.2 Logical Collapse: When Inference Masquerades as Claim

Interpretive overreach often occurs when the conditional status of an inference is lost during manuscript writing. A pathway enrichment result may begin as an observation, become an inference about possible pathway activity, and then be written as a mechanistic claim that the pathway “drives” or “mediates” a biological process. Similarly, a transcriptional cluster may first indicate a statistical grouping, then support a candidate cell‐state annotation, and finally be presented as a distinct functional cell type without sufficient validation.

The Observation‐Inference‐Claim framework is meant to keep these shifts visible in writing. At each step, the relevant question is whether the wording of the conclusion still matches the evidence actually in hand. Descriptive results may justify language such as “is enriched,” “is associated with,” or “suggests.” Stronger mechanistic language, including “drives,” “mediates,” or “is required for,” should be reserved for cases in which causal or functional evidence has been provided. This distinction is especially important when LLM‐assisted writing is used, because fluent summaries may smooth over uncertainty and make inferential statements appear more established than the evidence warrants.

## 4 Matching Claim Strength to Evidence Strength

To make the Observation‐Inference‐Claim framework more usable in practice, we propose an Evidence Ladder for matching the language of conclusions to the strength of supporting evidence (Table 1). The goal is not to assign rigid scores to studies, but to help authors, reviewers, and readers distinguish descriptive findings, biologically plausible interpretations, and mechanistic claims. Because single‐cell transcriptomic outputs are influenced by sampling, preprocessing, model choices, and validation depth, the strength of a claim should be aligned with both the breadth of data support and the type of evidence available [11, 28].

**TABLE 1:** Evidence Ladder for calibrating claim strength in single‐cell transcriptomic studies.

Evidence Level	Core Evidence Base	Recommended Language	Logical Boundary	Epistemic Status
L1: Descriptive	Clusters, DEGs, enrichment scores, inferred trajectories, proportion shifts	observed, detected, enriched, associated with	Restricted to data‐derived patterns in the analyzed dataset; avoid functional or causal wording unless additional evidence is provided	Descriptive statistical pattern
L2: Interpretative	L1 + marker genes, reference datasets, pathway knowledge, tissue context, cross‐dataset consistency, appropriate controls	suggests, is consistent with, is characterized by, may represent	Biologically plausible interpretation; alternative explanations should remain visible	Conditional biological inference
L3: Mechanistic	L2 + perturbation experiments, lineage tracing, orthogonal validation + protein‐level or spatial confirmation, functional assays	drives, mediates, is required for, is necessary/sufficient for	Requires direct testing of causal direction, necessity, sufficiency, or functional consequence within the relevant experimental context	Supported mechanistic claim

### 4.1 The Three Tiers of the Evidence Ladder

At the descriptive level, conclusions should remain close to data‐derived patterns. Cluster structures, DEGs, enrichment scores, inferred trajectories, and cell proportion shifts can justify verbs such as “observed,” “detected,” “identified,” or “enriched” [3, 11, 28]. These statements describe patterns present in the analyzed data, but they should avoid functional or causal implications unless additional evidence is provided.

At the interpretative level, statistical observations are combined with marker genes, reference datasets, pathway knowledge, tissue context, cross‐dataset consistency, and appropriate controls [18, 29, 30]. This level can support language such as “suggests,” “is consistent with,” “is characterized by,” or “may represent.” For example, marker‐supported annotation or pathway enrichment may suggest a candidate cell state or regulatory program, but such conclusions remain conditional and should acknowledge alternative explanations [26, 31]. Even when multi‐omic or pharmacological profiling is used to nominate therapeutic vulnerabilities, the appropriate claim is often that a candidate mechanism or vulnerability has been prioritized, not that it has been definitively established.

At the mechanistic level, stronger verbs such as “drives,” “mediates,” or “is required for” should be reserved for cases in which causal direction, necessity, sufficiency, or functional consequence has been directly tested [9, 27]. Perturbation experiments, lineage tracing, orthogonal validation, protein‐level or spatial confirmation, and functional assays can strengthen mechanistic claims, especially when performed in a relevant biological context [20, 21, 32, 33]. Even then, mechanistic conclusions should be framed according to the scope and limitations of the experimental system.

In practice, many studies occupy intermediate positions. Orthogonal measurements or ex vivo perturbations may strengthen a mechanistic interpretation without fully establishing endogenous necessity or sufficiency. These cases are best described as strengthened inferences or candidate mechanisms rather than definitive causal claims. The Evidence Ladder is therefore intended as a calibration tool: it encourages authors to choose verbs that reflect the level of evidence actually provided.

Table 2 uses a small set of published studies as schematic examples of how different evidence structures support different levels of interpretation. It is not intended as a scorecard, an exhaustive survey, or evidence about field‐wide prevalence of claim‐evidence mismatch. Rather, it shows how different evidence bases can support different claim strengths across observational atlases, computational inference studies, multimodal profiling, ex vivo functional assays, and perturbational designs. A systematic assessment of how often strong causal language is used without corresponding evidence would require a dedicated corpus‐level analysis, such as mapping mechanistic verbs to evidence types across a large set of publications. Although such an analysis is beyond the scope of this Comment, the framework proposed here could provide a basis for conducting it.

**TABLE 2:** Worked examples illustrating the application of the Evidence Ladder to selected single‐cell studies.

Study	Evidence structure	Evidence‐ladder position	Calibrated interpretation	Appropriate claim scope
Lim et al., 2024 [1]	Review of single‐cell and multi‐omics technologies	L1‐L2	Provides a broad overview of cell states, molecular profiling, and analytical opportunities	High‐resolution profiling expands descriptive and interpretative capacity, but does not by itself establish mechanism
Yuan et al., 2025 [24]	LINGER model + atlas‐scale multi‐omic data	L2	Prioritizes candidate gene regulatory links and regulatory programs	Computationally inferred networks remain predictive hypotheses until tested by perturbation or functional validation
Mennillo et al., 2024 [36]	Clinical cohorts + spatial multi‐omics	L2	Links treatment‐associated cellular compartments with spatial tissue states	Spatial proximity and association can nominate candidate cellular interactions, but do not alone establish mediation or functional driving
Wegmann et al., 2024 [37]	Ex vivo drug profiling + multi‐omic integration	L2‐L3 intermediate	Prioritizes therapeutic vulnerabilities and candidate resistance mechanisms	Ex vivo convergence strengthens mechanistic plausibility, but in vivo or endogenous functional validation is needed for stronger causal claims
Replogle et al., 2022 [20]	Genome‐scale Perturb‐seq	L3	Maps genetic perturbations to transcriptomic phenotypes	Perturbational evidence supports stronger functional claims within the tested experimental system

### 4.2 Epistemological Boundaries of Single‐Cell Transcriptomics

Single‐cell transcriptomics is particularly powerful as a discovery‐oriented approach [3, 4, 7]. It can generate hypotheses, characterize cellular heterogeneity, identify candidate cell states, and reveal molecular patterns that may not be apparent in bulk measurements. However, transcriptomic differences measured at single‐cell resolution are primarily associative [7, 11]. On their own, they usually cannot establish causal direction, functional necessity, or mechanistic sufficiency [9, 27].

This limitation does not reduce the value of single‐cell transcriptomic studies. Rather, it clarifies the level of conclusion that different evidence types can support. Descriptive analyses can identify patterns; integrative analyses can support biologically plausible interpretations [18, 26, 30, 31]; and perturbational, spatial, protein‐level, lineage‐tracing, or functional assays can strengthen mechanistic claims [21, 27, 32, 33, 34, 35]. Strong biological conclusions are entirely appropriate when the supporting evidence is strong enough to sustain them. Scientific writing should therefore make the status of each conclusion explicit, distinguishing what was observed, what is inferred, and what has been experimentally validated.

## 5 Conclusion

The rigor of single‐cell transcriptomic research depends not only on analytical sophistication, but also on the logical alignment between evidence and claims. Single‐cell transcriptomics excels at hypothesis generation, state discovery, and heterogeneity profiling, but static transcriptomic measurements are not, by themselves, sufficient to establish causal mechanisms. Strong mechanistic claims require corresponding validation.

In this Comment, we proposed an Observation‐Inference‐Claim framework and an accompanying Evidence Ladder to keep interpretation aligned with evidence. The aim is not to discourage biological interpretation, but to make clearer what has been observed, what is being inferred, and what has been functionally supported. As single‐cell technologies and LLM‐assisted scientific writing continue to expand, this calibration becomes increasingly important. The future of single‐cell transcriptomics will depend not only on higher‐resolution measurements, but also on more disciplined interpretation.

## Author Contributions

**Guangchuang Yu**: conceptualization, supervision, Writing – review and editing. **Shutong Lin**: writing – original draft, investigation, formal analysis.

## Conflicts of Interest

The authors declare no conflicts of interest.

## Data Availability

Data sharing not applicable to this article as no datasets were generated or analysed during the current study.
